# Boosting HIV Pre-exposure Prophylaxis (PrEP) Prescribing Confidence in a Southern Family Medicine Residency

**DOI:** 10.7759/cureus.93499

**Published:** 2025-09-29

**Authors:** Jonathan R Guin, Aaron B Stuber, Mary L Friend, Anne Halli

**Affiliations:** 1 Family, Internal, and Rural Medicine, The University of Alabama, Tuscaloosa, USA

**Keywords:** family medicine residency, hiv prevention, lgbtq medicine, pre-exposure prophylaxis (prep), rural family medicine

## Abstract

Introduction: Pre-exposure prophylaxis (PrEP) is a highly effective pharmacologic strategy for preventing HIV infection, yet it remains underutilized, particularly in the southeastern United States and among adolescent populations. Family medicine physicians, especially those practicing in rural areas, are well-positioned to identify and manage PrEP-eligible patients, including adolescents and members of LGBTQ+ (lesbian, gay, bisexual, transgender, and queer) communities. However, many providers report inadequate training in sexual health, LGBTQ+ care, and HIV prevention. This study aimed to assess the impact of a brief educational intervention on family medicine residents’ knowledge, attitudes, and skills in prescribing PrEP to a diverse range of high-risk individuals.

Methods: A one-hour educational session on PrEP was delivered at a rural-focused family medicine residency program in the southeastern US. Twenty residents completed pre- and post-intervention surveys consisting of true/false knowledge questions and five-point Likert-scale items assessing comfort with PrEP prescribing. Descriptive statistics and paired t-tests were used to evaluate change.

Results: While no statistically significant improvement was noted in knowledge scores (mean: 84.7% pre vs. 87.2% post, p = 0.108), participants reported notable increases in prescribing comfort. The proportion of residents who felt somewhat or extremely comfortable with initiating PrEP increased from six (30%) to 14 (72%), continuing therapy from seven (35%) to 14 (72%), and overall PrEP management from seven (35%) to 12 (62%). Eighteen residents (90%) identified limited knowledge as a barrier to PrEP prescribing, and several cited uncertainty about engaging LGBTQ+ patients in risk-based conversations.

Conclusions: A single, low-resource educational intervention can improve family medicine residents’ confidence in PrEP prescribing, despite minimal change in knowledge scores after this small-scale study. These findings underscore the importance of integrating sexual health, including prescribing PrEP for HIV, and LGBTQ+-inclusive training into residency education, particularly in high-need regions. Scalable educational models like this may help advance the *Ending the HIV Epidemic in the US* initiative by equipping primary care providers to engage more effectively in HIV prevention and address disparities among vulnerable populations.

## Introduction

The *Ending the HIV Epidemic in the US* (EHE) initiative, launched in 2019, aims to reduce new HIV infections by 75% by 2025 and by 90% by 2030 through strategic investment in prevention, diagnosis, and treatment efforts [1]. Family medicine physicians are well-positioned to play a pivotal role in achieving these goals, particularly by identifying and managing pre-exposure prophylaxis (PrEP)-eligible patients across the lifespan. Despite this potential, over 38,000 Americans are diagnosed with HIV annually. Hispanic/Latino and Black/African American individuals remain disproportionately affected, accounting for the majority of new diagnoses, with nearly half occurring in the Southern United States [2]. Among these cases, 70% result from male-to-male sexual contact and 22% from heterosexual contact [2].

PrEP comes in both once daily oral forms (emtricitabine/tenofovir disoproxil fumarate or emtricitabine/tenofovir alafenamide) and long-acting injectable forms (cabotegravir every two months or lenacapavir every six months). The emtricitabine/tenofovir combinations prevent HIV by inhibiting HIV reverse transcriptase. Cabotegravir is an integrase strand transfer inhibitor (INSTI), while lenacapavir is an HIV-1 capsid inhibitor. PrEP reduces the risk of HIV acquisition by more than 99% when taken daily [3], and the U.S. Preventive Services Task Force (USPSTF) recommends its use for all individuals at increased risk, including adolescents weighing at least 35 kilograms (kg) [4]. However, despite these clear guidelines, family medicine physicians in the South continue to have some of the nation’s lowest rates of PrEP prescribing [5,6]. Specifically, the Southern United States had the lowest prevalence of active PrEP prescriptions in the country at 29.8 per 100,000 population, compared with 62.3 in the Northeast, 36.0 in the West, and 30.1 in the Midwest. The South also had the lowest PrEP-to-need ratio (1.5), meaning only 1.5 people were on PrEP for every new HIV diagnosis, compared with 4.7 in the Northeast [6]. Given that family medicine physicians comprise a substantial proportion of primary care providers in the South, these figures underscore the region’s persistently low PrEP prescribing rates despite clear clinical guidelines. Frequently cited barriers include limited education on HIV prevention, discomfort with sexual history taking, stigma, and lack of confidence in managing patients at elevated risk, i.e., LGBTQ+ (lesbian, gay, bisexual, transgender, and queer) individuals [7-9].

These barriers are compounded by broader gaps in LGBTQ+-specific health training within family medicine education. Nationally, most family medicine residency programs offer minimal formal instruction in LGBTQ+ care, and many do not include PrEP counseling or sexual health beyond basic reproductive care [10,11]. Without this foundational exposure, providers may feel ill-equipped to conduct inclusive sexual histories, assess HIV risk in LGBTQ+ patients, or engage in culturally responsive prevention discussions. These deficits contribute to both missed opportunities for PrEP and lower engagement among at-risk populations.

Rural residency programs may face even greater challenges, as geographic isolation and limited institutional resources often restrict residents’ exposure to LGBTQ+ patients and access to faculty with HIV prevention expertise. Fewer clinical encounters with at-risk populations, scarce opportunities to work alongside infectious disease or sexual health specialists, and the absence of in-house PrEP prescribers can hinder skill development, leaving graduates less confident in providing comprehensive HIV prevention services [12]. Yet these are the very programs serving communities with the highest incidence of new infections [2]. Closing this gap requires scalable, practical educational strategies. Brief interventions, such as one-time lectures or case-based discussions, have shown promise in enhancing provider knowledge and comfort in underserved settings [13,14]. Even brief educational exposure, such as a single-session, iPad-based survey and PrEP knowledge assessment delivered at medical conferences and didactic lectures similar to this study, was associated with measurable gains in provider knowledge, which in turn correlated with higher reported rates of prior PrEP prescribing (21% overall; 34% among HIV providers vs. 9% among non-HIV providers) and increased intent to prescribe PrEP in the future (64% overall) [15].

This study evaluates the impact of a single, one-hour educational session on family medicine residents’ knowledge and prescribing comfort related to PrEP. Conducted at a rural-focused training program in the southeast United States, the intervention was designed as a low-resource model to assess feasibility and efficacy. We hypothesized that even a brief training could enhance provider readiness to offer PrEP, particularly in the context of limited prior education and LGBTQ+ training exposure. By addressing both clinical content and cultural competence, this study offers a practical approach to improving HIV prevention education in primary care.

## Materials and methods

Study design

This was a pre- and post-intervention study assessing changes in knowledge and prescribing comfort related to PrEP among family medicine residents in a rural-focused program in the southeastern United States. The purpose of this study is to determine if a single education session for family medicine residents could improve knowledge and prescribing skills for PrEP. A one-hour educational lecture was developed and delivered by the study authors. The session reviewed the epidemiology of HIV, clinical guidelines for initiating and maintaining PrEP from the CDC, and addressed provider concerns, including risk assessment, follow-up care, documentation, and billing. Our project was reviewed by the Office for Research Ethics & Compliance at the University of Alabama and was given an exemption from IRB review (IRB ID: 24-08-7844).

Inclusion and exclusion criteria

All family medicine residents who attended the grand rounds session or later viewed the recorded lecture were eligible to participate. Participants who failed to complete both the pre- and post surveys were excluded from the final analysis. A total of 29 residents attended the lecture, and 20 of them were included in the study.

Data collection

Data were collected anonymously via researcher-generated surveys administered through Qualtrics (Provo, UT) before and immediately after the session. Both pre- and post surveys included seven demographic questions, five Likert-scale items related to prescribing comfort ("extremely uncomfortable," "somewhat uncomfortable," "neither uncomfortable nor comfortable," "somewhat comfortable," and "extremely comfortable"), one question regarding barriers to prescribing PrEP, and 18 true/false PrEP prescribing knowledge questions (Appendix). The survey instrument was developed by the study authors based on CDC PrEP guidelines and existing literature on provider barriers to PrEP prescribing. Survey items were reviewed by faculty with expertise in PrEP and LGBTQ+ health to ensure content validity. Formal psychometric validation was not conducted due to the pilot nature of the study. Future iterations of the survey may benefit from broader validation across multiple residency programs.

Statistical analysis

Descriptive statistics were used to summarize participant demographics and baseline responses. A positive response was indicated by the two higher-scoring responses, "somewhat" or "extremely comfortable," while a negative response was indicated by the two lower-scoring responses, "extremely" or "somewhat uncomfortable." Paired t-tests were used to assess within-subject changes in knowledge. Participant responses for PrEP knowledge questions were matched between surveys using a participant-generated anonymous identifier code. Percentages are presented in n (%) format.

## Results

The study included 20 family medicine residents distributed across postgraduate year (PGY)-1 (25.0%), PGY-2 (25.0%), and PGY-3 (50.0%) levels. Participants identified primarily as female (55.0%) or male (45.0%), with no respondents identifying as non-binary, transgender, or other gender identities. The majority of participants were White/Caucasian (60.0%), followed by Asian (20.0%), African American (10.0%), and other racial or ethnic backgrounds (10.0%, including “Other” and “Prefer not to say”). Most residents identified as heterosexual (95.0%), with only one participant identifying as homosexual (5.0%). Table 1 summarizes the demographic data.

**Table 1:** Resident demographics summary (n = 20). PGY: postgraduate year.

	Category	n (%)
Resident level	PGY-1	5 (25.0%)
	PGY-2	5 (25.0%)
	PGY-3	10 (50.0%)
Gender identity	Male	9 (45.0%)
	Female	11 (55.0%)
	Non-binary	0 (0.0%)
	Trans-male	0 (0.0%)
	Trans-female	0 (0.0%)
	Other	0 (0.0%)
	Prefer not to say	0 (0.0%)
Race/ethnicity	African American	2 (10.0%)
	White/Caucasian	12 (60.0%)
	Asian	4 (20.0%)
	Other race	1 (5.0%)
	Prefer not to say	1 (5.0%)
Sexuality	Heterosexual	19 (95.0%)
	Homosexual	1 (5.0%)
	Bisexual	0 (0.0%)
	Other	0 (0.0%)
	Prefer not to say	0 (0.0%)

Following the brief educational intervention, participants demonstrated a slight overall improvement in PrEP-related knowledge on their post surveys. The average correct response rate increased from 84.7% pre intervention to 87.2% post intervention (p = 0.108), and while not statistically significant, it still represented a 2.5% absolute increase. While several questions showed no change due to already high baseline scores (e.g., questions 4, 6, 10, and 15 with 100% correct at both time points), notable improvements were observed in areas such as PrEP eligibility criteria (Q1-Q3) and risk/benefit discussion (Q11-Q13). The largest absolute gains were seen in question 2 (70% to 85%) and question 3 (60% to 75%), both reflecting enhanced understanding of clinical considerations in identifying appropriate PrEP candidates. A slight decline was observed in question 17 (85% to 75%). Results are summarized in Table 2. No significant differences were observed in knowledge scores across training levels.

**Table 2:** Pre and post knowledge assessment by question with percent change (n = 20). Notable gains were observed in pre-exposure prophylaxis (PrEP) eligibility criteria and risk/benefit discussions, while some items showed no change due to high baseline scores.

Question	Pre, n (%)	Post, n (%)	Percent change
Q1	13 (65.0%)	15 (75.0%)	10
Q2	14 (70.0%)	17 (85.0%)	15
Q3	12 (60.0%)	15 (75.0%)	15
Q4	20 (100.0%)	20 (100.0%)	0
Q5	18 (90.0%)	19 (95.0%)	5
Q6	20 (100.0%)	20 (100.0%)	0
Q7	15 (75.0%)	15 (75.0%)	0
Q8	18 (90.0%)	18 (90.0%)	0
Q9	15 (75.0%)	15 (75.0%)	0
Q10	20 (100.0%)	20 (100.0%)	0
Q11	16 (80.0%)	17 (85.0%)	5
Q12	18 (90.0%)	19 (95.0%)	5
Q13	18 (90.0%)	19 (95.0%)	5
Q14	18 (90.0%)	17 (85.0%)	-5
Q15	20 (100.0%)	20 (100.0%)	0
Q16	17 (85.0%)	17 (85.0%)	0
Q17	17 (85.0%)	15 (75.0%)	-10
Q18	16 (80.0%)	16 (80.0%)	0
Overall	305 (84.7%)	314 (87.2%)	2.5

**Figure 1:**
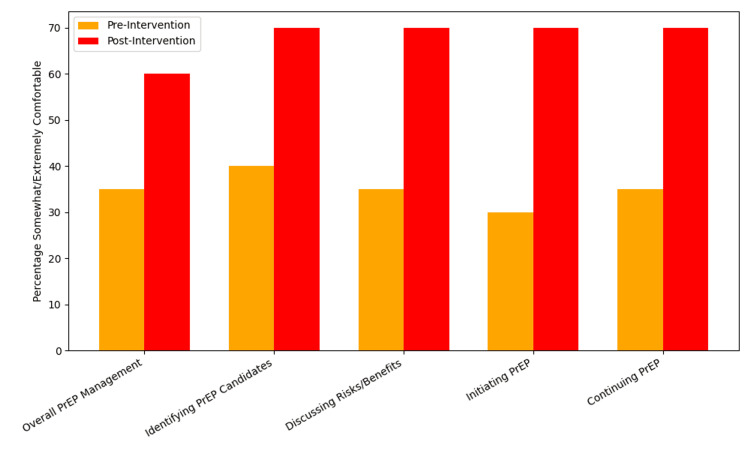
Comfort level with PrEP tasks: pre versus post intervention (n = 20). Marked increase from pre- to post intervention in the percentage of family medicine residents who are "somewhat" or "extremely comfortable" with tasks regarding prescribing PrEP. PrEP: pre-exposure prophylaxis.

As part of the pre- and post-intervention surveys, residents were asked about their comfort level regarding PrEP prescribing tasks. To provide more granular insight, Figure 1 displays item-level comfort ratings across all five Likert-scale domains. In contrast to the knowledge assessment, self-reported confidence in PrEP prescribing improved substantially following the educational session. The proportion of residents reporting being somewhat or extremely comfortable with overall PrEP management rose from seven (35%) to 12 (60%). Similar improvements were seen in identifying PrEP candidates from eight (40%) to 14 (70%), discussing risks/benefits of PrEP from seven (35%) to 14 (70%), initiating PrEP from six (30%) to 14 (70%), and continuing PrEP from seven (35%) to 14 (70%). These consistent gains across all items suggest broad-based improvement in clinical confidence following the one-hour intervention. Full pre- and post-survey responses regarding comfort levels are presented in Table 3 and Table 4, respectively.

**Table 3:** Pre-intervention prescribing comfort levels by task. Distribution of Likert-scale responses across five prescribing domains prior to the educational session. PrEP: pre-exposure prophylaxis.

Response category	Overall management	Identifying candidates	Discussing risks/benefits	Initiating PrEP	Continuing treatment
Extremely uncomfortable	3 (15.0%)	3 (15.0%)	2 (10.0%)	5 (25.0%)	4 (20.0%)
Somewhat uncomfortable	6 (30.0%)	4 (20.0%)	4 (20.0%)	7 (35.0%)	7 (35.0%)
Neither comfortable nor uncomfortable	4 (20.0%)	5 (25.0%)	7 (35.0%)	2 (10.0%)	2 (10.0%)
Somewhat comfortable	6 (30.0%)	7 (35.0%)	6 (30.0%)	5 (25.0%)	5 (25.0%)
Extremely comfortable	1 (5.0%)	1 (5.0%)	1 (5.0%)	1 (5.0%)	2 (10.0%)

**Table 4:** Post-intervention prescribing comfort levels by task. Marked increases in “somewhat” and “extremely comfortable” responses across all domains following the intervention. PrEP: pre-exposure prophylaxis.

Response category	Overall management	Identifying candidates	Discussing risks/benefits	Initiating PrEP	Continuing treatment
Extremely uncomfortable	0 (0.0%)	1 (5.0%)	1 (5.0%)	1 (5.0%)	1 (5.0%)
Somewhat uncomfortable	3 (15.0%)	1 (5.0%)	2 (10.0%)	2 (10.0%)	1 (5,0%)
Neither comfortable nor uncomfortable	5 (25.0%)	4 (20.0%)	3 (15.0%)	3 (15.0%)	4 (20.0%)
Somewhat comfortable	8 (40.0%)	8 (40.0%)	7 (35.0%)	11 (55.0%)	6 (30.0%)
Extremely comfortable	4 (20.0%)	6 (30.0%)	7 (35.0%)	3 (15.0%)	8 (40.0%)

Residents were also asked to select their self-identified barriers to prescribing PrEP with the following options: "I am unaware of PrEP," "My knowledge of PrEP is limited," "I didn't know primary care could prescribe PrEP," "I have concerns of patient coverage for PrEP," "I have concerns about the behavioral consequences of PrEP," "I have concerns about the health consequences of PrEP," "I have concerns patients will not adhere to PrEP," and "Other" with a field allowing free text. Notably, 18 (90%) participants identified “limited knowledge of PrEP” as a key barrier to prescribing, despite their relatively high baseline knowledge as seen on the pre-test. These results are summarized in Table 5.

**Table 5:** Self-identified barriers to prescribing PrEP (n = 20). PrEP: pre-exposure prophylaxis.

Barrier	Responses, n (%)
I am unaware of PrEP	1 (5.0%)
My knowledge of PrEP is limited	18 (90.0%)
I didn't know primary care could prescribe PrEP	0 (0.0%)
I have concerns about patient coverage for PrEP	7 (35.0%)
I have concerns about the behavioral consequences of PrEP	3 (15.0%)
I have concerns about the health consequences of PrEP	3 (15.0%)
I have concerns that patients will not adhere to PrEP	2 (10.0%)
Other	0 (0.0%)

No significant differences in knowledge or comfort scores were observed across PGY levels (PGY-1, PGY-2, PGY-3), suggesting the intervention was equally effective across training stages. These results suggest that even with minimal overall knowledge improvement, a brief one-hour educational intervention can substantially enhance provider confidence across key aspects of PrEP care.

## Discussion

Our findings contribute to a growing body of evidence suggesting that brief, targeted educational interventions can substantially improve provider comfort with PrEP prescribing. Although knowledge scores showed only a slight increase (84.7% to 87.2%, p = 0.108), participants reported substantial gains in self-reported confidence across key clinical domains, including initiating, continuing, and managing PrEP. These gains were reflected in absolute increases of 25% to 40% across key PrEP prescribing domains, indicating meaningful changes in perceived clinical confidence. This aligns with prior research demonstrating that confidence and willingness to prescribe are often more responsive to experiential and values-based learning than to didactic content alone [15].

Additionally, our study reflects persistent training gaps in both HIV prevention and LGBTQ+ health within family medicine residency education, with 90% of residents citing limited PrEP knowledge as a barrier, only 30% reporting comfort initiating PrEP, and just 35% comfortable with overall PrEP management at baseline. These findings align with national surveys indicating that over 70% of US family medicine residency programs provide fewer than five hours of LGBTQ+-focused curriculum, and fewer than half include formal PrEP prescribing education [10,11]. When included, such content is often unstandardized and may lack depth in key areas such as sexual history taking, PrEP prescribing, and adolescent LGBTQ+ health [11].

The expanded item-level analysis (Figure 1) provides greater granularity on where improvements occurred. Comfort in initiating and continuing PrEP both rose markedly from 30% to 70% and 35% to 70%, respectively, suggesting that residents felt more capable of managing PrEP across its continuum. Increases were also observed in identifying appropriate patients (from 40% to 70%) and in discussing risks and benefits (35% to 70%). These results were visualized in Figure 1, which clearly depicts the across-the-board gains in confidence. Notably, even the domain of overall PrEP management, which typically reflects more advanced decision-making, showed a meaningful increase (from 35% to 60%). Importantly, the detailed breakdown of comfort across individual prescribing tasks provides valuable insight for curriculum developers. It suggests that brief educational sessions can affect not only overall readiness but also specific prescribing behaviors. Also, this structure allows educators to identify which elements of PrEP care require additional reinforcement, including residents' lingering discomfort with follow-up management or risk-based discussions.

The implications of these gaps are significant. LGBTQ+ patients, especially gay and bisexual men, transgender individuals, and queer adolescents of color, remain among the highest-risk populations for HIV acquisition in the United States, yet PrEP access and uptake remain disproportionately low. Recent national data show that while Black and Hispanic/Latino men who have sex with men (MSM) account for the majority of new HIV diagnoses, they represent only 9% and 16% of PrEP users, respectively, and transgender women, despite high HIV prevalence, constitute less than 1% of all PrEP users [5]. Without training that explicitly prepares providers to deliver culturally competent, affirming care, these patients may not disclose behaviors relevant to HIV risk, or may avoid care altogether. Studies have shown that perceived provider stigma is a key deterrent to PrEP uptake, particularly among Black and Latino gay and bisexual men in the South [9]. Stigma is especially harmful to transgender individuals, contributing to long-standing disparities in health access and outcomes [16].

Integrating LGBTQ+-inclusive sexual health training into primary care residencies is therefore not only a matter of education but one of equity and trust-building. Implementation research shows that programs embedding PrEP into routine workflows, supported by faculty with lived experience or specialization in LGBTQ+ health, are more likely to normalize sexual health discussions and reduce provider bias [17,18]. Additionally, targeted interventions that include structured mentorship or community-based panels have demonstrated success in enhancing provider empathy and improving willingness to prescribe PrEP [19,20].

Rural programs, like the one in this study, face unique challenges. Faculty may lack experience with PrEP, and institutional support for LGBTQ+ education may be limited by geography, ideology, or funding [10]. Yet these settings often serve populations with the highest HIV burden and the fewest prevention resources. Our findings demonstrate that even a single, well-structured session can lead to meaningful improvements in provider confidence, suggesting a scalable model for high-impact change. Embedding such sessions within regular didactics, paired with mentorship and practical tools such as documentation templates, monitoring guides, and prescribing checklists, may further support sustainable change.

Several participants identified a lack of familiarity with PrEP guidelines as an ongoing barrier to prescribing. This highlights the importance of combining values-driven education with concrete, skills-based instruction on clinical workflows and follow-up care, particularly as national guidelines continue to emphasize proactive PrEP counseling and regular monitoring [21]. Future interventions may benefit from integrating real-world scenarios, LGBTQ+ patient vignettes, or contributions from community stakeholders to further enhance empathy, cultural humility, and practical readiness.

Our study has several limitations. The small sample size (n = 20) and single-institution design limit the generalizability of findings to broader residency populations, particularly those in urban or non-Southern settings. Additionally, the reliance on self-reported comfort may not accurately reflect actual clinical behavior or prescribing patterns. Future studies should incorporate objective measures such as electronic health record audits or patient-level outcomes to assess real-world impact. The absence of follow-up data also precludes assessment of long-term knowledge retention or sustained behavior change. Finally, the voluntary nature of participation may introduce selection bias, as residents more interested in LGBTQ+ health or HIV prevention may have been more likely to engage. As this was an exploratory pilot study with a small sample (n = 20), no formal power calculation was conducted. Findings should be interpreted as hypothesis-generating and may inform future larger-scale studies.

Taken together, our results emphasize that meaningful improvements in provider confidence can be achieved even with minimal resources, provided the intervention is relevant, inclusive, and practice-oriented. As the landscape of HIV prevention evolves, primary care physicians, particularly those in underserved and rural settings, must be equipped not only with up-to-date clinical knowledge but also with the cultural competence needed to engage diverse, high-risk populations. Educational models that blend concise content delivery with inclusive communication strategies represent a promising path forward. Future work should explore how residency programs can sustainably integrate PrEP education and LGBTQ+-inclusive care into their core curricula. Evidence suggests that provider training, mentorship, and structural support are strongly associated with improved PrEP prescribing behavior [17,22] and are an essential next step as we strive toward the goals of the *Ending the HIV Epidemic* initiative.

## Conclusions

This study shows that a brief, low-resource educational session can improve family medicine residents’ confidence in prescribing PrEP, even though knowledge scores changed only slightly. Conducted in a rural-focused southeastern program, the intervention demonstrates both the feasibility and importance of incorporating PrEP training into residency curricula in areas with high HIV incidence and low prescribing rates. The session also addressed ongoing gaps in LGBTQ+-inclusive sexual health education, which is essential for reducing stigma and supporting patient engagement. While our findings are limited by a small sample size and a single-site design, the results suggest that short, practical interventions can make a meaningful impact on provider readiness. Embedding these sessions into regular didactics, along with mentorship and practical tools, may help sustain improvements. Larger, multi-institutional studies are needed to confirm generalizability and evaluate long-term impact. Preparing residents with both clinical skills and cultural competence for PrEP prescribing is an important step toward the goals of the *Ending the HIV Epidemic in the US* initiative.

## Appendices

**Table 6:** Pre- and post-lecture survey. Credits: Aaron B. Stuber et al. PrEP: pre-exposure prophylaxis; T/F: True/False; HIV: human immunodeficiency virus.

Question	Text	Category
1	What is your current role?	Demographics
2	What is your specialty?	Demographics
3	What is your current academic class?	Demographics
4	Please indicate your age in years.	Demographics
5	Which of the following best describes your gender identity?	Demographics
6	Which of the following best describes your race/ethnicity?	Demographics
7	Which of the following best describes your sexual orientation?	Demographics
8	How comfortable are you with the overall management of pre-exposure prophylaxis (PrEP) for HIV?	Attitudes
9	How comfortable are you at identifying patients who would benefit from pre-exposure prophylaxis (PrEP) for HIV?	Attitudes
10	How comfortable are you at discussing the risks and benefits of pre-exposure prophylaxis (PrEP) for HIV?	Attitudes
11	How comfortable are you at initiating pre-exposure prophylaxis (PrEP) for HIV?	Attitudes
12	How comfortable are you with continuing stable treatment of pre-exposure prophylaxis (PrEP) for HIV?	Attitudes
13	What are some barriers you face to prescribing pre-exposure prophylaxis (PrEP) for HIV?	Attitudes
14	A patient must be HIV-negative to take pre-exposure prophylaxis (PrEP).	T/F Knowledge
15	Patients taking pre-exposure prophylaxis (PrEP) should have a follow-up HIV test at 3-month intervals.	T/F Knowledge
16	If a patient seroconverts (becomes HIV-positive) while taking pre-exposure prophylaxis (PrEP), they may continue taking PrEP.	T/F Knowledge
17	Infectious disease physicians are the only practitioners who can prescribe pre-exposure prophylaxis (PrEP).	T/F Knowledge
18	With daily dosing, pre-exposure prophylaxis (PrEP) is >90% effective in preventing HIV infection.	T/F Knowledge
19	There is limited scientific evidence to support the use of pre-exposure prophylaxis (PrEP) for HIV prevention.	T/F Knowledge
20	A patient with an undetectable HIV viral load can take pre-exposure prophylaxis (PrEP).	T/F Knowledge
21	Pre-exposure prophylaxis (PrEP) is known to cause kidney failure in a majority of patients who take it.	T/F Knowledge
22	A patient with a detectable HIV viral load can take pre-exposure prophylaxis (PrEP).	T/F Knowledge
23	Pre-exposure prophylaxis (PrEP) may be used by men who have sex with men to prevent HIV.	T/F Knowledge
24	Pre-exposure prophylaxis (PrEP) may be used by people who inject drugs to prevent HIV.	T/F Knowledge
25	Pre-exposure prophylaxis (PrEP) is not effective for transgender and gender non-conforming people to prevent HIV.	T/F Knowledge
26	Pre-exposure prophylaxis (PrEP) is only used by men who have sex with men.	T/F Knowledge
27	Adolescents (age 13-18) with risk factors for HIV can take pre-exposure prophylaxis (PrEP).	T/F Knowledge
28	There is no risk of HIV from vaginal sex.	T/F Knowledge
29	New HIV infections occur disproportionately in men who have sex with men.	T/F Knowledge
30	The incidence of HIV diagnoses in Black men who have sex with men is higher than for any other group of people.	T/F Knowledge
31	A majority of prescriptions for pre-exposure prophylaxis (PrEP) are given to transgender women who have sex with men.	T/F Knowledge
